# Fabrication and errors in the bibliographic citations generated by ChatGPT

**DOI:** 10.1038/s41598-023-41032-5

**Published:** 2023-09-07

**Authors:** William H. Walters, Esther Isabelle Wilder

**Affiliations:** 1 Mary Alice & Tom O’Malley Library, Manhattan College, Riverdale, NY USA; 2 Department of Sociology, Lehman College, The City University of New York, Bronx, NY USA; 3 Doctoral Program in Sociology, CUNY Graduate Center, The City University of New York, New York, NY USA

**Keywords:** Information technology, Software

## Abstract

Although chatbots such as ChatGPT can facilitate cost-effective text generation and editing, factually incorrect responses (hallucinations) limit their utility. This study evaluates one particular type of hallucination: fabricated bibliographic citations that do not represent actual scholarly works. We used ChatGPT-3.5 and ChatGPT-4 to produce short literature reviews on 42 multidisciplinary topics, compiling data on the 636 bibliographic citations (references) found in the 84 papers. We then searched multiple databases and websites to determine the prevalence of fabricated citations, to identify errors in the citations to non-fabricated papers, and to evaluate adherence to APA citation format. Within this set of documents, 55% of the GPT-3.5 citations but just 18% of the GPT-4 citations are fabricated. Likewise, 43% of the real (non-fabricated) GPT-3.5 citations but just 24% of the real GPT-4 citations include substantive citation errors. Although GPT-4 is a major improvement over GPT-3.5, problems remain.

## Introduction

ChatGPT-3 (Chat Generative Pre-trained Transformer 3), released to the public in November 2022 by OpenAI, uses elements of artificial intelligence—including natural language processing (NLP), machine learning, and deep learning—to produce or alter texts in ways that mimic human writing or speech. Among other things, ChatGPT can respond to specific or open-ended questions; engage in conversation; summarize, translate, or edit text provided by the user or included in its information base; and generate original text based on the user's instructions^1^. Its ability to generate short reports and papers has led to concerns regarding educational and academic integrity^2–9^.

It is important to realize, however, that ChatGPT is fundamentally not an information-processing tool, but a *language*-processing tool. It mimics the texts—not necessarily the substantive content—found in its information base^10,11^. ChatGPT has also been known to “hallucinate”—to provide factually incorrect responses—although OpenAI reports that this is less of a problem with ChatGPT-4, released in March 2023, than with earlier versions of the software^12–15^.

This study investigates one particular type of hallucination: fabricated bibliographic citations that do not correspond to actual scholarly works. For 84 documents generated by GPT-3.5 and GPT-4, we determine the percentage of the 636 cited works that are fabricated rather than real, along with the percentage of the works (articles, chapters, books and websites) for which the larger publication or organization (journal, book, publisher) is also fabricated. For the citations that correspond to real works, we also assess the prevalence of various citation errors (e.g., incorrect author names, article titles, dates, journal titles, volume numbers, issue numbers, page numbers, and publisher/organization names). Finally, we briefly investigate citation formatting errors and the characteristics of the real and fabricated hyperlinks included in ChatGPT citations.

### Significance of the topic

Understanding the nature and extent of fabricated citations and citation-related errors is important for at least three reasons. First, we can help maintain scientific integrity by raising awareness of these problems and minimizing investigators' tendency to trust ChatGPT more than they should. Although authors are ultimately responsible for their work, we routinely (and justifiably) place confidence in software and hardware without double-checking the results; we don't manually recalculate the output of statistical software, for instance. That same level of trust is not appropriate with generative AI tools, however, since the tasks performed by AI are fundamentally different. Researchers must learn the extent to which we can trust these tools, then revise that assessment as the technologies improve and our expectations change.

Second, we can help students learn by showing them the strengths and limitations of software such as ChatGPT. Students who are knowledgeable about fabricated citations will presumably be more likely to take the literature review process seriously and to do the work themselves—or to check and build on their ChatGPT citations in ways that lead them to accomplish many of the intended learning goals. As discussed later, citation errors may also help faculty identify cases in which tools such as ChatGPT have been used.

Third, an understanding of fabricated citations and errors can help AI developers improve the software and the methods used for development and testing. As we suggest in the “Discussion” section, citations are a special type of text for which predictive word choice, paraphrasing, and related techniques may be detrimental rather than useful. For instance, the replacement of a title word with a more common, or even more appropriate, synonym reduces the value of the title as a search string and as a unique identifier of a particular cited work.

### Previous research

Several authors have noted that ChatGPT tends to cite works that do not actually exist^12,16–25^, and 6 studies have systematically investigated the extent of the problem^13,15,26–29^. For instance, Wagner and Ertl-Wagner^15^ used GPT-3.5 to answer 88 questions in 8 subspecialty areas of radiology, then evaluated the citations included in those responses. Of the 343 citations, 64% were fabricated (i.e., could not be found in PubMed or on the open web). As Table 1 shows, the proportion of fabricated citations is typically in the 47–69% range, with a higher rate in geography than in medicine. Across all 6 studies, 51% of the 732 citations were fabricated. (By *citations* we mean bibliographic references—the works cited at the end of each paper—rather than the individual parenthetical citations or note numbers that appear throughout the text.)

**Table 1:** Studies that have evaluated the extent to which ChatGPT produces fabricated citations.

Study	ChatGPT version	Discipline	Papers	Citations	Percentage fabricated (%)
Day^28^	3.5	Geography	5	18	94
Gravel et al.^13^	3.5	Medicine	20	59	69
Wagner and Ertl-Wagner^15^	3	Radiology	88	343	64
Hueber and Kleyer^29^	4?	Medicine	3	19	58
Bhattacharyya et al.^27^	3.5	Medicine	30	115	47
Athaluri et al.^26^	3	Medicine	50	178	17

For Athaluri et al.^26^, the *percentage fabricated* value refers to journal articles. If we include book chapters and websites, the value may be as high as 23%.

Accurate citations provide evidence in support of claims, allow readers to evaluate that evidence, establish a context for new empirical work, and draw attention to gaps in the research literature^18^. Fabricated citations work against each of those goals, especially since (a) ChatGPT's fabricated citations tend to look legitimate at first glance^13,28^ and (b) ChatGPT often provides incorrect responses when asked “Is this citation correct” or “Do you fabricate citations?”^12,13,18,24,29^.

Two studies have investigated the errors in non-fabricated ChatGPT citations. Bhattacharyya et al.^27^ found that 87% of the citations to real (non-fabricated) works had 1 or more of 7 errors—incorrect PubMedID number, author name, article title, date, journal title, volume number, or page numbers—and Day^28^ reported very similar results. Errors in the numerical components of citations are especially common^26,27^.

## Methods

We used GPT-3.5 and GPT-4 to generate short papers on 42 multidisciplinary topics, then compiled data on the 636 bibliographic citations found in the 84 papers. With that information, we searched multiple databases and websites to evaluate (a) the proportion of fabricated citations, (b) the prevalence of errors in the citations to non-fabricated papers, (c) the extent of adherence to the fundamentals of APA citation format, and (d) the characteristics of the hyperlinks found in the ChatGPT citations. Supplementary Appendix 1 presents the 42 paper topics. Supplementary Appendix 2 includes the 84 texts generated by GPT-3.5 and GPT-4. Supplementary Appendix 3 is the resulting data file.

### Paper topics and prompts

GPT-3.5 and GPT-4 were each used to generate 42 short papers (literature reviews) of the kind typically expected of students in first-year composition courses at U.S. universities. The 42 paper topics include the health effects of e-cigarettes, the unintended consequences of China's one-child policy, the potential use of cloning to bring back extinct species, the original purpose of Stonehenge, the economic and political impact of global metal shortages, the effects of Brexit on the U.K. economy, the relationship between self-efficacy and self-reported assessments of ability, the historical origins of the concept of purgatory, and the advantages and disadvantages of molten salt reactors for nuclear power production. The predominance of broad, Wikipedia-style overviews; the avoidance of specialized scientific subjects; and the focus on social, political, and environmental topics are all typical of the papers submitted for first-year composition courses. For the complete list of topics, see Supplementary Appendix 1.

A new chat/conversation was initiated for each paper topic, and each was embedded within a prompt of the type recommended by Atlas^30^. The same introductory text was used in each case: “I want you to act as an academic researcher. Your task is to write a paper of approximately 2000 words with parenthetical citations and a bibliography that includes at least 5 scholarly resources such as journal articles and scholarly books. The paper should respond to this question: ‘[paper topic].’”.

Because the ChatGPT response field is limited in length, the system's initial response to each prompt was always less than 2000 words and never a complete paper. An additional prompt of “Please continue” was used, sometimes more than once, to get ChatGPT to continue the text exactly where it had left off. If “Please continue” was entered near the end of the paper or within the bibliography, ChatGPT sometimes provided supplementary text (with or without additional bibliographic references), presumably on the assumption that the original response was unsatisfactory or inadequate. For this study, any text that followed the initial bibliography was not regarded as part of the paper generated by ChatGPT and was therefore excluded from the analysis. Supplementary Appendix 2 includes the complete texts generated in the first week of April 2023 by GPT-3.5 and GPT-4 in response to each of the 42 prompts (paper topics).

### Data compilation and analysis

For each paper, we recorded the length of the text, the number of works listed in the bibliography, and any notable irregularities (e.g., obvious misinformation or fabricated empirical results, parenthetical citations without corresponding entries in the list of references).

The 84 papers include 636 citations (cited works). For each, we recorded full bibliographic information, the number of times the work was cited in the text, whether the work was scholarly or popular, and the type of publication: article, book, chapter, or website. The *website* category includes only web content other than articles, books, and chapters.

We then searched Google, Google Scholar, Amazon, the Directory of Open Access Journals, PubMed, Scopus, WorldCat, publishers' and journals' websites, and other sources to determine whether each cited work was real or fabricated. Our searches included all the search methods and databases mentioned in previous studies of ChatGPT citations^15,26,27^.

We regarded a cited work as *real* (non-fabricated) if we found an actual work that was a match or near-match with regard to both title and author(s). That is, we allowed for the possibility that the work was real but the citation was not quite correct. An incorrect journal title for a real article was regarded as a citation error—not as evidence of a fabricated work. As a final check on each apparently fabricated work, we browsed the relevant journal volume/issue and used the search function on the publisher's website to verify that no such work existed.

For each non-fabricated work, we identified any substantive errors in the bibliographic information provided by ChatGPT—incorrect authorship, title, journal, publisher, volume number, pagination, etc. This part of the evaluation did not include formatting errors such as irregularities in capitalization, punctuation, or order of the bibliographic elements, but it did include errors and omissions that might lead to difficulty in finding or retrieving the full text. When identifying errors, we disregarded the publisher and edition statements of 12 early monographic works (e.g., *Summa Theologica*).

For both real and fabricated works, we evaluated the degree of adherence to the fundamental features of APA citation format—i.e., whether any bibliographic elements were missing or presented incorrectly. (Unless instructed otherwise, ChatGPT uses APA format.) This last evaluation did include some APA-specific elements such as title capitalization and the use of authors' initials rather than full names, but we also checked for elements that are common to nearly all citation formats, such as the inclusion of publisher/organization names. While an incorrect publisher name was counted as a substantive error, the absence of a publisher name was counted as a formatting error. We did not account for the absence of italics, which are not used in ChatGPT output, or for deviations from APA format with regard to place of publication, state abbreviations, or the inclusion of issue numbers, since these elements have varied with recent editions of the APA Publication Manual.

Finally, we recorded whether each citation included a hyperlink and whether the link led to the cited work.

## Results

Although we asked for papers of approximately 2000 words, none of the 84 papers are more than 1400 words long, and most are substantially shorter (Table 2). As other authors have noted^30^, GPT-3.5 works best with short, conversational responses, and this is true of GPT-4 as well. Follow-up questions can be used to generate additional text, however, and simply typing “Continue” will often achieve the same result. Text generated after the initial response may not be fully integrated into the initial text, however, and the subsequent responses will include additional bibliographic citations only about 20% of the time.

**Table 2:** Characteristics of the papers generated by ChatGPT.

	GPT-3.5	GPT-4
Number of papers	42	42
Mean length, in words	834	1003
SD of length, in words	128	190
Minimum length	596	638
Maximum length	1207	1397
Mean number of cited works	5.3	9.9
SD of number of cited works	1.9	3.0
Minimum number of cited works	2	5
Maximum number of cited works	11	17
Percentage with 5 or more cited works	71%	100%

Although we asked for at least 5 bibliographic citations, 12 of the 42 GPT-3.5 papers cite fewer than 5 works. Each of the GPT-4 papers does cite at least 5 works, however. Every cited work is at least broadly relevant to the paper topic, and 93% are scholarly rather than popular works.

### Extent of fabrication

Of the 222 works cited in the GPT-3.5 papers, 55% are fabricated (Table 3). That is, they do not exist as actual works that have been published, presented, posted, or otherwise publicly disseminated. The articles and book chapters cited by GPT-3.5 are more likely to be fabricated than real, while the cited books and websites are more likely to be real.

**Table 3:** Extent of fabrication among the works cited in the ChatGPT papers.

	GPT-3.5	GPT-4
Percentage of cited works that are fabricated (and number of cited works, in parentheses)		
All works	55% (222)	18% (414)
Articles	73% (128)	18% (255)
Books	23% (78)	8% (126)
Chapters	70% (10)	70% (23)
Websites	50% (6)	10% (10)
Among fabricated works, percentage of		
All works for which the larger work/org. is fabricated	5%	5%
Articles for which the journal is fabricated	2%	2%
Books for which the publisher is fabricated	0%	0%
Chapters for which the book is fabricated	57%	19%
Website for which the organization is fabricated	0%	0%

Unlike the GPT-3.5 citations, most of the GPT-4 citations refer to works that are verifiably real. Only 18% are fabricated. Even with GPT-4, however, 70% of the cited book chapters are fabricated.

Both GPT-3.5 and GPT-4 seem to have special difficulty with book chapters. As Table 3 shows, most of the fabricated article, book, and website citations include the names of real journals, publishers, and organizations. In contrast, many of the fabricated references to book chapters mention books that do not themselves exist; neither the chapters nor the books are real.

ChatGPT often provides inaccurate responses when asked to verify the legitimacy of the works it cites^12,13,18,24,29^. Interestingly, however, one of the 84 works generated for this study (GPT-3.5, topic 28) includes a caveat within the text of its response: “These are sample bibliography entries and are not meant to represent actual sources”. Another (GPT-3.5, topic 19) recommends that the user cite more than the 5 references provided in response to the prompt.

### Substantive citation errors

Among the GPT-3.5 cited works that are real (not fabricated), 43% have one or more substantive citation errors: incorrect author name(s), article titles, dates, journal titles, volume/issue/page numbers, or publishers (Table 4). More than a third of the articles have incorrect volume, issue, or page numbers, and 22% of the cited works have incorrect dates (years). With older works, a common problem is the reporting of online posting dates rather than the original publication dates. There are relatively few incorrect titles or author names, however, and most of those discrepancies are minor—e.g., attaching the initials of one author to the next author on the list. (As described in the “Methods” section, we regarded minor errors in titles or authorship as citation errors rather than as evidence of fabricated citations.)

**Table 4:** Substantive citation errors in the citations to the real (non-fabricated) works.

	GPT-3.5	GPT-4
Number of real (non-fabricated) cited works	101	340
Percentage with 1 or more substantive citation errors	43%	24%
Percentage with incorrect author name(s)	14%	6%
Percentage with incorrect title of the work itself	6%	3%
Percentage with incorrect date	22%	16%
Percentage of articles with incorrect journal title	14%	4%
Percentage of articles and chapters with incorrect volume, issue, or page numbers	34%	13%
Percentage of books, chapters, and websites with incorrect publisher or organization	9%	3%

Except as indicated, these values refer to all works. There is no meaningful variation among the publication types (articles, books, chapters, and websites).

Just as GPT-4 has fewer fabricated citations than GPT-3.5, it also has fewer substantive citation errors (Table 4). Again, incorrect numeric values—volume/issue/page numbers and years of publication—are the most common problems. Fewer than 7% of the GPT-4 citations have even minor errors in their authors, titles, journal titles, or publishers.

### Formatting errors and hyperlinks

With both GPT-3.5 and GPT-4, every citation is in APA format. However, more than 40% of the citations have minor formatting errors (Table 5). The most common error, by far, is improper title capitalization (e.g., capitalizing all the words in an article title). No other type of error appears in more than 8% of the GPT-3.5 citations or in more than 2% of the GPT-4 citations. Overall, the real citations and the fabricated citations display the same kinds of formatting errors.

**Table 5:** Citation formatting errors in the real (non-fabricated) and fabricated works.

	GPT-3.5	GPT-4
Number of cited works	222	414
Percentage with 1 or more citation formatting errors	45%	40%
Percentage with improper title capitalization	44%	39%
Percentage with unnecessary quotation marks	8%	0%
Percentage with author first name(s) rather than initials	4%	0%
Percentage with incorrect version of author name(s)	1%	0%
Percentage of articles and chapters lacking page numbers	1%	2%
Percentage of books, chapters, and websites lacking publisher/organization name	2%	1%

Except as indicated, these values refer to all works. There is no meaningful variation among the publication types (articles, books, chapters, and websites) or between fabricated and non-fabricated works.

Contrary to APA style, very few of the ChatGPT article citations include hyperlinks (Table 6). GPT-3.5 and GPT-4 each provide links for fewer than 10% of their citations to real works, and links are actually more likely to be found within the fabricated citations. When links *are* included within the citations to real works, about one-third of them are inaccurate. GPT-4 performs only slightly better than GPT-3.5 in this regard.

**Table 6:** Links included in ChatGPT citations.

	GPT-3.5	GPT-4
Percentage of real works for which the citation includes a link		
All works	5%	8%
Articles	6%	7%
Books	0%	3%
Chapters	0%	0%
Websites	100%	100%
Percentage of those works for which the link leads to the cited work		
All works	60%	70%
Articles	50%	64%
Books	–	100%
Chapters	–	–
Websites	67%	67%
Percentage of fabricated works for which the citation includes a link		
All works	18%	8%
Articles	18%	9%
Books	6%	0%
Chapters	14%	6%
Websites	100%	100%

## Discussion

In terms of both fabricated citations and citation errors, GPT-4 is a major improvement over GPT-3.5. Within this set of documents, 55% of the GPT-3.5 citations but just 18% of the GPT-4 citations are fabricated. Likewise, 43% of the real GPT-3.5 citations but just 24% of the real GPT-4 citations include substantive citation errors. Our results are broadly consistent with previous research (Table 1).

Because detailed information on the use of ChatGPT is not available, we cannot know what proportion of users are taking advantage of the enhanced performance of GPT-4. As of August 2023, GPT-3.5 is freely available online while GPT-4 is available only to paid subscribers; the individual rate is US $20 per month.

### Why do fabricated citations persist?

Despite the improved performance of GPT-4, the fundamental question remains: Why does ChatGPT generate fabricated citations at all? Bhattacharyya et al.^27^^,p. 6^ assert that the difficulty is inherent in large language models, which "use deep neural networks to predict the next word in a sequence of text and provide responses based on statistical patterns learned during training…. As such, ChatGPT cannot distinguish between accurate and false information." If ChatGPT relied solely on predictive algorithms to generate citation information, however, we might expect *all* the bibliographic citations to be fabricated or otherwise incorrect. Our experience with ChatGPT suggests that the software may attempt to recognize bibliographic citations and to treat them differently than regular text—e.g., to copy them exactly rather than predicting/paraphrasing in the usual manner. Fabricated citations may therefore represent an inability to fully recognize which specific parts of the text should be treated as bibliographic data.

Sanchez-Ramos et al.^22^ suggest that “the causes for the inaccuracies of ChatGPT are related to the vast amount of text data from diverse sources and inconsistency errors or inaccuracies in the primary data”. This argument is not entirely convincing, since other tools that rely on data harvested from non-standardized texts (e.g., Google Scholar) have not had such high fabrication or hallucination rates. It is true, however, that inconsistencies in the source documents may explain why ChatGPT seems to have trouble distinguishing between the various dates reported for each paper, such as the dates of preprint posting, final-version posting, and official publication. Google Scholar has had similar difficulties^31–33^. Bibliographic problems may persist to the extent that citations are treated as regular text rather than a type of text for which special processing is required.

### Implications of these findings

As noted in the Introduction, a better understanding of fabricated citations can help researchers uphold scientific integrity and help students understand the importance of identifying, evaluating, and citing relevant literature. Users of ChatGPT are cautioned to check the citations it generates—and, of course, to evaluate the quality of the cited works themselves. At least two of the citations fabricated by GPT-3.5 for this study—Supplementary Appendix 3, citations 11.02 and 19.02—are to journals whose publishers have been identified as predatory^34^.

Journal editors and publishers may also want to ensure that fabricated citations do not find their way into the scholarly literature. A recent paper suggests that while this is unlikely to be a problem with the final versions of published articles, fabricated citations are more likely to appear in the papers posted to preprint servers and institutional repositories^35^.

Instructors tasked with detecting AI-generated text—in undergraduate research papers, for instance—may find it helpful to examine the works cited in those papers. To our knowledge, no publicly available AI text detector checks for fabricated citations when evaluating documents, but the presence of fabricated citations is a distinctive characteristic of ChatGPT text. Likewise, the citation errors generated by ChatGPT—numeric errors, in particular—are also distinctive. Although the best AI detectors are highly accurate^36–40^, each advance in generative AI technology requires a corresponding improvement in AI detection capabilities.

Finally, our investigation confirms that even with the latest version of ChatGPT, misinformation can be found throughout the generated texts—not just in the reference lists. Although we made no systematic attempt to detect false statements, a quick reading of the documents revealed that both GPT-3.5 and GPT-4 continue to generate assertions that are obviously incorrect. (See, for example, topic 28 in Supplementary Appendix 3.) Moreover, five of the “literature reviews” generated by ChatGPT are structured and presented as empirical studies, with fabricated methods and results. One of them (GPT-4, topic 16) even includes fabricated correlation coefficients, regression coefficients, and *p* values. These errors are potentially dangerous, and they are exacerbated by the fact that ChatGPT often stands by its incorrect statements when asked to verify them^12,13,18,24,29^. As Gravel et al.^13^ have pointed out, ChatGPT is “confidently wrong” in its incorrect assertions. This may be because ChatGPT is fundamentally a text transformer—not an information retrieval system—and because it is designed to repeat behaviors that result in favorable human feedback^41,42^. Humans are more likely to be satisfied with confident responses^43–45^, so the AI provides confident responses, correct or otherwise.

### Supplementary Information

Supplementary Information 1.

Supplementary Information 2.

Supplementary Information 3.

## Data Availability

All the data are provided in the Supplementary Appendices. Supplementary Appendix 1 lists the 42 paper topics. Supplementary Appendix 2 presents the 84 texts generated by GPT-3.5 and GPT-4. Supplementary Appendix 3 presents the data compiled for the analyses.
