# Supplementary material for: Fabrication and errors in the bibliographic citations generated by ChatGPT
Source: Sci Rep. 2023 Sep 7;13:14045. doi: 10.1038/s41598-023-41032-5 (PMC10484980; doi:10.1038/s41598-023-41032-5)
Supplement: Supplementary file 1 — Supplementary Information 1. [file 41598_2023_41032_MOESM1_ESM.docx]

Fabrication and errors in the bibliographic citations generated by ChatGPT

**Supplementary Appendix 1: ChatGPT paper topics**

ChatGPT-3.5 and ChatGPT-4 were each used to generate papers on 42 topics: 8 in the humanities (topics 1–8), 25 in the social sciences (topics 9–33), and 9 in the natural sciences (topics 34–42).

1. Why was Stonehenge built? What are the most likely explanations, and what evidence supports or challenges each of them?

2. What were the causes of the Second Boer War (1899 to 1902)? What did the British Empire, the South African Republic, and the Orange Free State each hope to achieve?

3. What major 19th-century literary works received initially negative reviews but are now regarded as key contributions to literature? What accounts for the changing opinions of these works?

4. What studies best demonstrate how quantitative methods can be applied to the analysis of English-language literary works?

5. When did unicorns first appear in literature? How has the depiction of unicorns and their characteristics changed over time?

6. Will languages other than English gain importance over time as languages of scientific discourse?

7. What accounts for the dominance of American and British songwriters and musicians in 20th- and 21st-century popular music? Why did no other countries' artists have a similar impact?

8. What are the historical origins of the religious concept of purgatory? Who put forth the concept of purgatory? Was it accepted initially? When and how did it assume its place within Catholic theology?

9. Among retired Americans and those approaching retirement, are there distinct types of migration or geographic mobility (distinct groups of migrants)? What are the distinctive characteristics of each type or group?

10. What were the unintended effects of China's one-child policy? How have the Chinese government and the Chinese people responded to them?

11. How have ride-sharing services such as Uber and Lyft influenced overall employment in the taxi and ride-sharing industry? How have they influenced wages?

12. In the present-day U.S., what are the most effective strategies by which wealthy individuals can minimize their income tax payments?

13. What are the long-term economic and political impacts of the global shortages of copper, lithium, nickel, and cobalt?

14. What is the best way to determine the impact of Brexit on the UK economy?

15. Why did the U.S. government first institute minimum wage laws? What were they hoping to achieve?

16. Among American college students, to what extent do self-reported assessments of ability represent self-efficacy rather than ability?

17. Can synchronous demonstrations, delivered online, be just as effective as in-person lab instruction for undergraduate biology courses?

18. Can the educational success of U.S. charter schools at the high school (secondary) level be attributed to factors other than the socioeconomic characteristics of their students?

19. Do students who get free meals in grades P–5 do better academically than students of similar backgrounds who do not get free meals?

20. Is there evidence to support the idea that high school math teachers who struggled with math can be more effective than those for whom math came easily?

21. To what extent are university students' evaluations of their instructors related to the difficulty of the course? What is the best way to overcome any bias related to the link between teaching evaluations and course difficulty?

22. What are the advantages and disadvantages of taking a "gap year" of employment or volunteer work between high school and college—for individuals and for society?

23. Are there systematic differences in the organizational leadership styles of men and women? To what extent are they unique to either women or men?

24. Who were the most successful businesswomen of the 20th century?

25. Internationally, how have Patrick S. Atiyah's "Accidents, Compensation and the Law" and "The Damages Lottery" influenced legal education, practice, and theory?

26. What are the military missions or situations for which aerial drones have proven most successful? In what areas do they have the greatest unmet potential?

27. What occupations are most likely to disappear entirely over the next 20 years?

28. In the U.S., what safety-related innovations (devices, policies, or procedures) were once mandated by law or regulation but later abandoned? Why were they abandoned? On what grounds should safety-related innovations be evaluated?

29. Do the fans at a football stadium influence the outcome of the game? Can we isolate the impact of the fans' behavior from the impact of having home-field advantage (and more fans in the stadium)?

30. Across nations, what is the influence of gun control legislation on rates of gun-related homicide, suicide, and accidental death? What factors make these comparisons potentially difficult?

31. Are adolescents who play violent video games especially likely to commit acts of violence? Do violent video games have other negative (or positive) psychological effects?

32. In terms of recruiting, training, and managing personnel, what are the most effective methods of preventing police violence against the public ("police brutality")?

33. What percentage of political assassination attempts are successful? What evidence can be used to address this question?

34. At the individual level, what is the impact of professional dental care on morbidity and mortality risk?

35. How harmful are e-cigarettes to the health of those who use them, relative to conventional cigarettes?

36. To what extent do sleep disorders influence the productivity of the American labor force?

37. Can cloning or similar methods be used to bring back extinct plant species? Extinct animal species?

38. What strategies have proven most effective as methods of stabilizing and increasing the orangutan population?

39. To what extent can global climate change be attributed to ruminant grazing and dairy farming?

40. What is the best way to gauge the environmental impact of a large-scale switch to electric vehicles for private passenger transportation in the United States? Account for the impact of the vehicles themselves as well as the need to generate electricity from sources such as natural gas, coal, nuclear, wind, and hydropower.

41. Which island nations and coastal nations will be most affected by climate change? What steps are they taking to prepare?

42. How are molten salt reactors different from conventional nuclear fission reactors? What are their unique advantages and disadvantages? In what ways are they more or less safe than conventional fission reactors?

Although most of these topics were suggested by personal experience with students and their written work [1], we also consulted about two dozen web sites for potential paper topics. Topics 24, 33, and 42 are similar to those suggested by Paperell.net [2], while topics 19, 22, and 37 are similar to those suggested by Sarikas, Allison, and Kearney [3–5], respectively.

The original version of topic 12—"In the present-day U.S., what are the most effective strategies by which wealthy individuals can avoid paying income tax?”—was not accepted by GPT-3.5, which responded, "I'm sorry, but I cannot fulfill this request. As an AI language model developed by OpenAI, I am programmed to avoid engaging in unethical or illegal behavior, including assisting with tax evasion or facilitating illegal activities. Additionally, writing an academic paper that promotes tax evasion would go against the principles of responsible and ethical research." Adding “(but legal and ethical)” before “strategies” led to essentially the same response. The third version of the question, which replaced "avoid paying income tax" with "minimize their income tax payments,” was accepted by GPT-3.5. That version of the prompt was used in the analyses.

For topic 29, we had American football in mind, but both GPT-3.5 and GPT-4 interpreted the question in terms of association football (soccer).

**References for Supplementary Appendix 1**

1. Walters, W.H. *et al.* A multi-method information literacy assessment program: Foundation and early results. *Portal: Libraries and the Academy* **20**, 101–135 (2020).

2. Paperell.net. 200 best research paper topics for 2023 + examples. https://paperell.net/blog/best-research-paper-topics (2023).

3. Sarikas, C. 113 great research paper topics. *PrepScholar* https://blog.prepscholar.com/good-research-paper-topics (25 Jan. 2020).

4. Allison, N. 250+ interesting research paper topics for 2022. *MyPerfectWords* https://myperfectwords.com/blog/research-paper-guide/research-paper-topics (16 Mar. 2023).

5. Kearney, V. 100 technology topics for research papers. *Owlcation* https://owlcation.com/academia/100-Technology-Topics-for-Research-Paper (26 Oct. 2022).
